# Evaluation of the Safety and Immunogenicity of Duck-Plague Virus *gE* Mutants

**DOI:** 10.3389/fimmu.2022.882796

**Published:** 2022-04-20

**Authors:** Yaru Ning, Yalin Huang, Mingshu Wang, Anchun Cheng, Renyong Jia, Mafeng Liu, Dekang Zhu, Shun Chen, Xinxin Zhao, Shaqiu Zhang, Qiao Yang, Ying Wu, Juan Huang, Bin Tian, Xumin Ou, Sai Mao, Qun Gao, Di Sun, Yanlin Yu, Ling Zhang

**Affiliations:** ^1^ Research Center of Avian Disease, College of Veterinary Medicine, Sichuan Agricultural University, Chengdu, China; ^2^ Institute of Preventive Veterinary Medicine, Sichuan Agricultural University, Chengdu, China; ^3^ Key Laboratory of Animal Disease and Human Health of Sichuan Province, College of Veterinary Medicine, Sichuan Agricultural University, Chengdu, China

**Keywords:** duck plague virus, gE, extracellular domain, genetic stability, pathogenicity, vaccine

## Abstract

Duck plague (DP) is an acute infectious disease in the duck industry. The duck plague virus (DPV) is the pathogen, a subfamily of *alphaherpesvirinae*. gE is a type I membrane protein that contains three parts: an extracellular domain, a transmembrane domain, and a cytoplasmic domain. gE is the major virulence determinant of α-herpesvirus. However, the functions of the gE extracellular and cytoplasmic domains have not been reported in DPV. In this study, a gE extracellular domain deletion mutant and a gE cytoplasmic domain deletion mutant were constructed from DPV. Virus replication kinetics showed that the growth titers of both the gE ectodomain-deleted mutant virus and the gE cytoplasmic domain-deleted virus in DEFs were lower than that of the parental virus CHv-50. DPV CHv-gEΔET and DPV CHv-gEΔCT were continuously passed to the 20th passage in DEFs and the 10th in ducklings. The mutant virus DNA after passage was extracted for identification. The results showed that the gE ectodomain and gE cytoplasmic domain deletion mutant viruses have good genetic stability. The ducklings in each group (n=10) were inoculated with the same titers of DPV CHv-gEΔET, DPV CHv-gEΔCT, DPV CHv-ΔgE, and parental CHv-50, respectively. Clinical symptoms and serum antibody levels were detected after inoculation. The results showed that the virulence of DPV CHv-gEΔCT to ducklings was reduced compared with parental CHv-50, while the virulence of DPV CHv-gEΔET to ducklings was significantly reduced. 10^5^ TCID_50_ DPV CHv-gEΔET or DPV CHv-ΔgE can induce ducklings to produce DPV-specific antibodies, protect the ducklings from virulent CHv challenge. Therefore, DPV CHv-gEΔET may serve as a promising vaccine candidate to prevent and control duck plague.

## Introduction

Duck plague (DP) is an acute disease with a high fatality rate caused by DPV, which has caused significant economic losses to the duck industry around the world and has shown a trend of younger age in recent years (1–3). Studies have shown that duck herd immunization with the attenuated vaccine can prevent the occurrence of duck plague, but after immunization, it is impossible to distinguish whether it is wild virus infection or vaccine immunization produces antibodies, resulting in the lack of strong physicochemical technical support for the technical plan to purify DPV infection through immunization (4). Therefore, there is an urgent need to develop a more effective vaccine to eradicate DPV. It has been reported that many countries have successfully eradicated pseudorabies through the gene deletion DIVA (differentiating infected from vaccinated animals) vaccine. Like PRV, DPV is also a member of the *alphaherpesviruses* (5–7). Therefore, the emerging DPV gene-deleted attenuated mutant virus is promising as a candidate vaccine for the control and eradication of duck plague.

DPV is a member of the *alphaherpesvirus* subfamily, with a double-stranded DNA genome of approximately 162 kb and a capsid, tegument, and envelope. The gE protein in DPV is encoded by the *US8* gene and has the characteristics of a typical type I membrane protein. The polypeptide chain transmembrane once, the N-terminus is outside the cell membrane, and the C-terminus is inside the cell membrane. Therefore, according to the transmembrane structure of gE protein, we divided it into three parts: extracellular domain, transmembrane domain and intracellular domain. gE is a non-essential structural protein that facilitates the spread of viruses from cell to cell, under certain circumstances, promotes anterograde transport of latent virions after reactivation, and is neurotoxic (8). The viral plaques of the mutant virus BAC-CHv-ΔgE on DEFs were about 60% smaller than that of the wild virus BAC-CHv (9). Electron microscopy results showed that the deletion of DPV gE caused a large number of capsids to accumulate around the vesicles, and only a few were able to bud into vesicles, which is consistent with reports in other herpesviruses such as HSV-1, HSV-2, VZV, PRV, suggesting that gE plays an important role in virion morphogenesis prior to final cytoplasmic nucleocapsid wrapping (10, 11). The gE CT is involved in the second coating of the nucleocapsid in specific parts of the Golgi apparatus, selectively distributing nascent virions to cell junctions and promoting the spread of viruses between cells. Viruses with deletions in different regions of gE CT were constructed on HSV-1 gD gene deletion strains, and it was found that after deletion of amino acids 470-495, a large number of nucleocapsids accumulated in the cytoplasm (12). DPV gE CT interacts with UL11. In the absence of gE CT, the amount of UL11 packaged into virions is reduced by 58.1 ~ 80%, and the nucleocapsid cannot complete the secondary coating to form complete virions, which inhibits the release of the virus (13, 14). gE ET mainly plays a role in the intercellular propagation between epithelial cells and polar cells such as neural tissue. Through the receptor mechanism, gE ET localizes the gE/gI complex at the extracellular junction and binds to the adjacent cell receptor, promoting the fusion of infected cells and adjacent non-infected cells. The amino acids 277, 291, and 348 of HSV gE ET were mutated to construct three mutant strains. Small plaques were formed after infecting cells, similar to the *gE* gene deletion strain, and the transmission of the virus from the cornea to the epithelial tissue was restricted (15). The deletion of amino acids 208-236 in the cysteine region of VZV gE ET changed the distribution of gE on the cell membrane and affected the spread of the virus between cells (16).

Notably, DPV can replicate and persist at high levels in duck tissues (17), which is associated with the ability of the virus to evade host immune defenses. gE forms a dimer with gI and participates in the immune evasion function of the virus to enhance the virulence of the virus. It is a good vaccine target protein. PRV gE has been reported to inhibit cGAS/STING-mediated IFN-β production by degrading CBP to disrupt the enhanced assembly of IRF3 and CBP (18). HSV gE binds to the Fc segment of IgG, which spatially prevents IgG or Fc-dependent effector cells from approaching the virus or virus-infected cells. Complement Clq cannot bind to the IgG Fc site, blocking the classical pathway of complement (19, 20). Protects viruses from immune processes such as antibody-dependent cytotoxicity and antibody-dependent cell-mediated phagocytosis (21–23). gE can also interact with prohibitin-1, which is conserved in all herpesviruses, for cell-to-cell transmission through the MAPK/ERK pathway (24). However, the molecular mechanism by which DPV gE plays a role in cell-to-cell transmission, evading the immune responses, and enhancing viral virulence has not been fully elucidated. The reported virulence genes mainly express non-essential envelope glycoproteins or nonstructural proteins. For example, the main virulence genes of PRV include gB, gC, TK, US3 (25–27). Therefore, studying the function of DPV gE from histopathology and *in vivo* colonization is crucial for the prevention and treatment of DPV infection, and there is no report on the key regions of gE virulence genes in DPV.

This study constructed CHv-gEΔET and CHv-gEΔCT deletion mutant viruses using bacterial artificial chromosome cloning of the DPV CHv-BAC-G strain. The efficacy of the mutant virus as a candidate vaccine for the control or eradication of duck plague in duck flocks was evaluated, and the safety and immunogenicity of the mutant virus were evaluated.

## Materials and Methods

### Animals and Ethics Statement

9-day-old Cherry Valley duck embryos and 7-day-old healthy Cherry Valley ducks were purchased from a farm operated by Sichuan Agricultural University (Sichuan, China). All experimental ducks did not contain DPV and were negative for DPV antibodies (28). The experimental animal protocol was approved by the Ethics and Animal Welfare Committee of Sichuan Agricultural University and carried out following the Chinese version of the Guide for the Care and Use of Laboratory Animals.

### Viruses and Cells

Parent virus CHv-50 (GenBank: JQ647509.1) (3) and DPV CHv-ΔgE deletion mutant virus were provided by our laboratory. DEFs were prepared from 9-day-old Cherry Valley duck embryos in Modified Eagle’s Medium (MEM, Gibco, Rockford, USA) supplemented with 10% neonatal bovine serum (NBS, Gibco, Rockford, USA) and 1% antibiotics (penicillin and streptavidin), cultured at 37°C in a 5% CO_2_ incubator.

### Generation of Mutant Viruses DPV CHv-gEΔET and DPV CHv-gEΔCT

The DPV CHv-BAC-GS1783 (29) strain is stocked in our lab, in which the entire DPV CHv genome tagged with an enhanced green fluorescent protein (EGFP) is inserted BAC for stable propagation in *E. coli* strain GS1783. The construction of DPV CHv-gEΔET was based on two markerless two-steps Red recombination (30). Briefly, the linear PCR product, ‘a-*I-Sce I*-Kan-a-b’ was amplified and electroporated into DPV CHv-BAC-GS1783 to induce the first step of Red recombination, thereby replacing the *gE-ET* gene with Kan, a and b are the 40 bp homology arms on the left and right sides of the *gE-ET* gene, respectively. Subsequently, the *I-Sce I* site is cleaved by the *I-Sce I* endonuclease, and the Kan is removed in the second step by Red recombination. Amplify the linear PCR product again, ‘a-’UL23-a’-’UL23-b’-b-*I-Sce I*-Kan-b-c’, electroporate it into DPV CHv-BAC-gEΔET-GS1783, induce the first step of Red recombination, a, b and c are successive homology arms downstream of the miniF sequence. Subsequently, the *I-Sce I* site is cleaved by the *I-Sce I* endonuclease, and the Kan is removed in the second step by Red recombination. The deletion of gE-ET was confirmed by identifying primers and sequencing. The plasmid DPV CHv-gEΔET-GS1783 was transfected into DEFs, and purified by spotting to obtain the DPV CHv-gEΔET deletion mutant virus. The same procedure was performed to construct and obtain DPV CHv-gEΔCT deletion mutant virus. All primers used in this study are listed in **Table 1**.

**Table 1:** The primers used to construct and identify deletion-mutant virus CHv-gEΔET and CHv-gEΔCT.

Primer name	Sequence (5′-3′)	Purpose
ΔET⁃Kan⁃F	CTGCCGGCCAGACTACGGAACCTCAACAATTGGTACGATGTAGGGATAACAGGGTAATCGATTT	Replacement of the *gE ET* gene by the kan cassette
ΔET⁃Kan⁃R	TAATAGTCACGACCCCTAGTACTCCGAGACCGACTACAAACATCGTACCAATTGTTGAGGTTCCGTAGTCTGGCCGGCAGGCCAGTGTTACAACCAAT	
ΔCT⁃Kan⁃F	CGTGACTATTATAATCCTGGCTGTTTCATCCATCTTTTTATAGGGATAACAGGGTAATCGATTT	Replacement of the *gE CT* gene by the kan cassette
ΔCT⁃Kan⁃R	CTATTTCACTAGTGAGTCATTAGTTCAACATCCATGATCATAAAAAGATGGATGAAACAGCCAGGATTATAATAGTCACGGCCAGTGTTACAACCAAT	
gE-F gE⁃R	TCTCAAGACGCTCTGGAATC AGCGAGTACTTCTCTGCGTC	Identification of the *gE* gene deletion
BAC⁃Kan⁃F	TTATTAATCTCAGGAGCCTGTGTAGCGTTTATAGGAAGTAGTGTTCTGTCATGATGCCTGCAAGCGGTAACGAAAACGATTGTTACAACCAATTAACC	Delete BAC miniF sequence and EGFP selection marker
BAC⁃Kan⁃R	ATCGTTTTCGTTACCGCTTGCAGGCATCATGACAGAACACTACTTCCTATTAGGGATAACAGGGTAATCGAT	
BAC⁃UL23⁃F	GCCTGCAAGCGGTAACGAAAACGATTCAATTAATTGTCATCTCGG	Delete BAC miniF sequence and EGFP selection marker
BAC⁃UL23⁃R	CCGCTCCACTTCAACGTAACACCGCACGAAGATTTCTATTGTTCCTGAAGGCATATTCAACGGACATATTAAAAATTGA	
UL30-F	GGACAGCGTACCACAGATAA	Identification of the DPV *UL30* gene
UL30-R	ACAAATCCCAAGCGTAG	
UL23-F	GCCTGCAAGCGGTAACGAAAACGATTCAATTAATTGTCATCTCGG	ldentification of the *UL23* gene deletion
UL23-R	CCGCTCCACTTCAACGTAACACCGCACGAAGATTTCTATTGTTCCTGAAGGCATATTCAACGGACATATTAAAAATTGA	
BAC⁃F	GTTATCCACTGAGAAGCGAACG	Identification of the BAC miniF sequence deletion
BAC⁃R	GGCTGTAAAAGGACAGACCACA	

### Identification of Deletion Mutant Virus by PCR and Western Blot

After DPV CHv-ΔgE, DPV CHv-gEΔET, and DPV CHv-gEΔCT deletion mutant viruses infected DEFs to produce 80% lesions, the samples were frozen and thawed three times, and the obtained viruses were identified by PCR and Western blot. In PCR identification, primer UL30-F/R is used to identify whether the DPV UL30 gene is contained. Primers BAC-F/R are used to identify whether it contains miniF element, primer UL23-F/R is used to identify whether continuous *UL23* gene is contained, Primers gE-F/R were used to identify deletions of the *gE* gene. DEFs were infected with DPV CHv-ΔgE, DPV CHv-gEΔET, and DPV CHv-gEΔCT deletion mutant viruses with an MOI of 0.1 to analyze the gE expression in deletion mutant viruses, and 48 h later, cells were lysed with RIPA lysate, and the protein lysate was collected. Add 1% PMSF to the lysis buffer. Western blot analysis was performed with rabbit anti-gE and goat anti-rabbit IgG antibodies.

### Viral Growth Curves

Multistep growth kinetics of the parental strain CHv-50, DPV CHv-gEΔET, and DPV CHv-gEΔCT deletion mutant viruses were performed as previously described with minor modifications (31, 32). Briefly, DEFs cultured in 24-well cell culture plates were infected with a virus at an MOI of 0.01. After the virus was adsorbed for 2 h at 37°C and 5% CO_2_, the medium was discarded, the cells were washed with PBS (pH 7.4), and then the culture medium was replaced with MEM containing 2% NBS. The infected cells were collected at 12, 24, 48, and 72 h after infection, the volume of each sample was increased to 500 μL with MEM, the samples were freeze-thawed three times, and virus titers were determined by TCID_50_ on DEFs. All experiments were repeated 3 times.

### Evaluation of Genetic Stability of Deletion Mutant Viruses

DEFs were separately infected with the DPV CHv-ΔgE, DPV CHv-gEΔET and DPV CHv-gEΔCT deletion mutant viruses. After the cells appeared 80% lesions, the samples were frozen and thawed 3 times, and the first-generation viruses were collected. The virus was re-infected with DEFs to prepare the next generation of the virus, and the deletion mutant virus was uploaded to the 20th passage in DEFs according to this method. DNA was extracted from the 10th and 20th passages of each virus according to the instructions of the Magen kit, and PCR identification was performed. The primers used are listed in **Table 1**. Virus titers at passages 1, 5, 10, 15, and 20 for each virus were determined by TCID_50_ on DEFs. All experiments were repeated 3 times.

Forty 14-day-old ducklings were divided into 4 groups, the first 3 groups were inoculated with DPV CHv-ΔgE, DPV CHv-gEΔET, DPV CHv-gEΔCT deletion mutant virus by intramuscular injection at a dose of 10^6^ TCID_50_/dose, and the last group was intramuscularly inoculated with the same volume of MEM. Seven days after inoculation, 5 ducklings in each group were randomly selected for culling, and the pathological changes of liver, spleen, and duodenum were observed. Take 1 g of liver tissue from each group for grinding, add normal saline at a ratio of 1:9 to make a homogenate, and inoculate new 14-day-old ducklings again. In this way, the mutant virus is transmitted to the 10th generation in ducklings. DNA was extracted from the 1st, 5th, and 10th passages of each virus according to the instructions of the Magen kit, and PCR identification was performed. The primers used are listed in **Table 1**. TaqMan-MGB probe fluorescence real-time quantitative PCR method (33) was used to detect the viral copies in liver tissue at passages 1, 5, and 10 of each virus.

### Safety Evaluation of Deletion Mutant Viruses

130 14-day-old ducklings were divided into 13 groups. Groups 1, 2, and 3 were inoculated with CHv-50 by intramuscular injection at 10^4^ TCID_50_/dose, 10^5^ TCID_50_/dose, and 10^6^ TCID_50_/dose, respectively. Groups 4, 5, and 6 were inoculated with DPV CHv-ΔgE by intramuscular injection at 10^4^ TCID_50_/dose, 10^5^ TCID_50_/dose, and 10^6^ TCID_50_/dose, respectively. Groups 7, 8, and 9 were inoculated with DPV CHv-gEΔET by intramuscular injection at 10^4^ TCID_50_/dose, 10^5^ TCID_50_/dose, and 10^6^ TCID_50_/dose, respectively. Groups 10, 11, and 12 were inoculated with DPV CHv-gEΔCT by intramuscular injection of 10^4^ TCID_50_/dose, 10^5^ TCID_50_/dose, and 10^6^ TCID50/dose, respectively. The control group was intramuscularly inoculated with the same volume of MEM. Five ducks were randomly selected from each group to measure the rectal body temperature every day, and the death of each group of ducks was recorded for 10 days.

### Evaluation of Immune Efficacy of Deletion Mutant Viruses

40 14-day-old ducklings were divided into 4 groups, the first group was inoculated with a dose (10^7.7^ copies)/dose of live duck plague vaccine CVCC AV1222, and the second and third groups were inoculated with the same number of copies/dose of DPV CHv-ΔgE and DPV CHv-gEΔET deletion mutant virus, the fourth group was the control group, each inoculated with 1 mL of MEM. On the 14th day after immunization, the ducklings were injected with 100 LD_50_/duck virulent Chinese strains of duck plague virus (CHv) and were continuously observed for 10 days after the challenge, and the clinical symptoms and death were recorded every day.

Similar to the above grouping and inoculation, blood was collected from each group of ducklings on the 7th, 14th, 21th, and 28th days after immunization, and serum was collected for neutralizing antibody detection. The serum to be tested was filtered with a 0.22 filter and inactivated at 56°C for 30 min. The treated serum to be tested was diluted 2^1^ ~ 2^8^ times, and 50 µL per well was added to a 96-well plate with DEFs. Add 50 µL of 1000 TCID_50_ of CHv to each well, incubate at 37°C for 2 h, discard the supernatant and add 100 µL of MEM with 2% NBS. Continue to culture to observe cytopathic changes and calculate the neutralization index according to the Read-Muench method. All experiments were repeated 3 times.

### Statistical Analysis

The data are expressed as the means ± S.D. Statistical analysis was performed with Student’s *t*-test (GraphPad Prism 6); *P<0.05, **P<0.01, ***P<0.001 and ****P<0.0001 indicate statistical significance compared with the control.

## Results

### Construction and Rescue of CHv-gEΔET and CHv-gEΔCT Deletion Mutant Viruses

**Figure 1:**
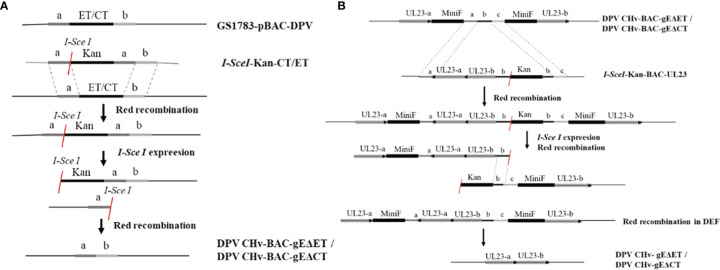
Homologous recombination diagram. Schematic diagram of the construction of the deletion mutant virus CHv-gEΔET and CHv-gEΔCT using the Red recombination system. **(A)** Schematic diagram of the construction of the deletion mutant virus CHv-BAC-gEΔET and CHv-BAC-EΔCT using the Red recombination system. **(B)** Schematic diagram of the construction of the deletion mutant virus CHv-gEΔET and CHvg EΔCT using the Red recombination system again.

**Figure 2:**
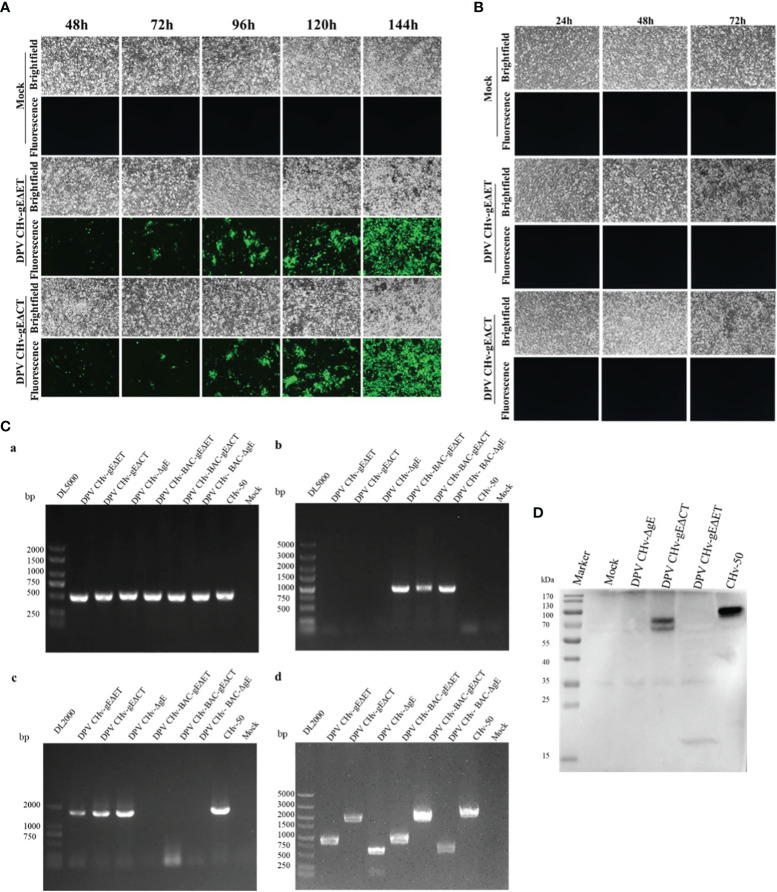
Generation and identification of mutant viruses for DPV CHv-gEΔET and DPV CHv-gEΔCT. **(A)** Transfection of the plasmids DPV CHv-gEΔET-GS1783/DPV CHv-gEΔCT-GS1783 into DEFs resulted in numerous fluorescent spots and cytopathies, the mutant virus DPV CHv-gEΔET/DPV CHv-gEΔCT were rescued. **(B)** Cells with cytopathic but no fluorescent spots were picked into new DEFs, resulting in the non-fluorescent mutant virus. **(C)** PCR identification of mutant viruses: **(a)** *UL30* gene identification primer, **(b)** BAC identification primer, **(c)** *UL23* gene identification primer, **(d)** *gE* gene deletion identification primer. **(D)** DEFs were infected with DPV CHv-ΔgE, DPV CHv-gEΔCT, DPV CHv-gEΔET, and DPV CHv-50, and an anti-gE polyclonal antibody was used for WB.

To explore the virulence gene functions of the ET and CT of gE, in DPV CHv-BAC-GS1783, we used two-step homologous recombination, the first homologous recombination first deleted gE-ET (**Figure 1A**), and constructed a DPV CHv-BAC-gEΔET-GS1783, the miniF was deleted by the second homologous recombination to construct DPV CHv-gEΔET-GS1783 (**Figure 1B**). DPV CHv-gEΔCT-GS1783 was constructed in the same way as described in Materials and methods. The plasmids of DPV CHv-gEΔET-GS1783 and DPV CHv-gEΔCT-GS1783 were respectively extracted and transfected into DEFs. At 48 h after transfection, small green fluorescent spots were observed in DEFs. After 144 h, green fluorescent spots were distributed in the entire field of view, and the cells produced lesions (**Figure 2A**). After collecting the virus solution to infect DEFs, pick the virus solution with cytopathic but no fluorescence to re-infect DEFs, resulting in stable DPV CHv-gEΔET and DPV CHv-gEΔCT deletion mutations with cytopathic and no fluorescence virus (**Figure 2B**).

### Identification of DPV CHv-gEΔET and DPV CHv-gEΔCT Deletion Mutant Viruses

To identify the non-fluorescent deletion mutant virus after purification, viral DNA was extracted from DEFs infected with DPV CHv-gEΔET, DPV CHv-gEΔCT, DPV CHv-ΔgE, and CHv-50 viruses, and PCR identification analysis were performed. Using the *UL30* gene identification primers can amplify the *UL30* gene fragment from the above DNA, indicating that the purified deletion mutant virus is duck plague virus, using the BAC identification primers cannot amplify the miniF element from the above DNA, indicating that all the purified deletion mutant virus has completely removed the miniF element, the *UL23* gene fragment can be amplified from the above DNA using the *UL23* gene identification primer, indicating that the purified deletion mutant virus *UL23* gene has been restored, amplification fragment of 884 bp, 1859 bp, 599 bp and 2072 bp from DNA infected with DPV CHv-gEΔET, DPV CHv-gEΔCT, DPV CHv-ΔgE and CHv-50 using *gE* gene deletion identification primers, respectively, indicating that the ET segment and CT segment of the *gE* gene have been correctly deleted, the sizes of the above target fragments were in line with expectations. DPV CHv-ΔgE and CHv-50 were used as negative and positive controls, respectively, and the Mock group was used as a control to demonstrate the specificity of the primers (**Figure 2C**).

The expression of gE protein in the deleted mutant virus was further analyzed by Western blot. As shown in **Figure 2D**, only CHv-50 can express the complete gE protein, about 20 kDa of gE protein in DPV CHv-gEΔET, DPV CHv-gEΔCT of gE protein is about 70 kDa, DPV CHv-ΔgE does not express gE protein, the results are in line with expectations, indicating that both DPV CHv-gEΔET and DPV CHv-gEΔCT recombinant viruses can be used in subsequent experiments.

### Growth Curves of Deletion Mutant Viruses

**Figure 3:**
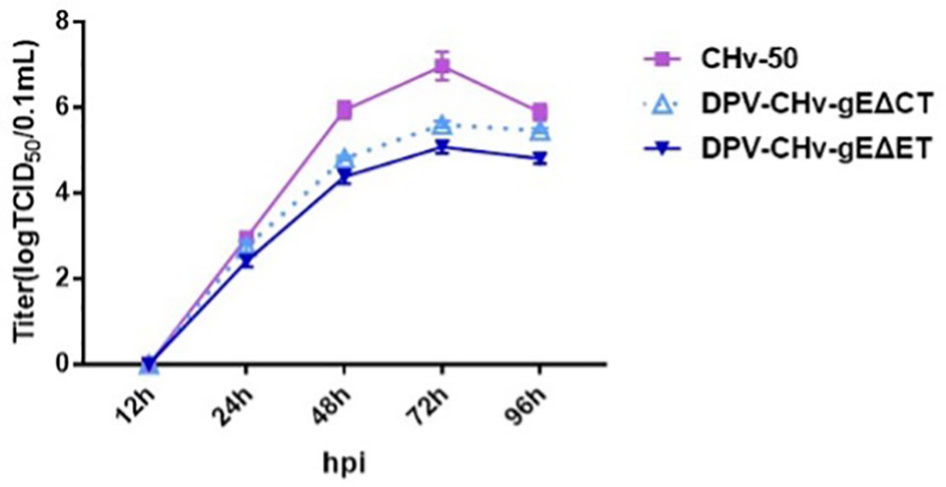
Determination of viral titers in growth kinetics. DEFs in 24-well plates were infected with 0.01 MOI of DPV CHv-gEΔCT, DPV CHv-gEΔET, and DPV CHv-50. Samples were collected at the indicated time points, and viral titers were determined. The data were presented as the mean ± standard deviation(SD) of three independent experiments.

The multi-step growth kinetics of DPV CHv-gEΔET and DPV CHv-gEΔCT deletion mutant viruses were evaluated after infecting DEFs with the same MOI = 0.01, collecting virus fluids at different time points, and detecting virus titers. As shown in **Figure 3**, the incubated virus entered the cell and started virus replication. At the initial 12 h of infection, the intracellular virus titers of the two deletion mutant viruses and the parental virus were not detected, indicating that the infectivity virus particles had not yet formed at this time. At 24 h after infection, infectious virions could be detected, indicating that a new generation of virions had been replicated in the cells. Then the virus titer gradually increased and reached a peak at 72 h, during which time the three virus strains were in the stage of replication and release. At 72 ~ 96 h after infection, the virus titers of the three virus strains all showed a downward trend. At this time, the virus titers decreased due to severe cytopathic changes. Compared with the parental virus, the viral titers of DPV CHv-gEΔET and DPV CHv-gEΔCT decreased by approximately 80 and 25 fold, respectively, at 72 h of infection. These results suggest that deletion of both gE-ET and gE-CT affects the proliferative capacity of the virus.

### Genetic Stability of DPV CHv-gEΔET and DPV CHv-gEΔCT

**Figure 4:**
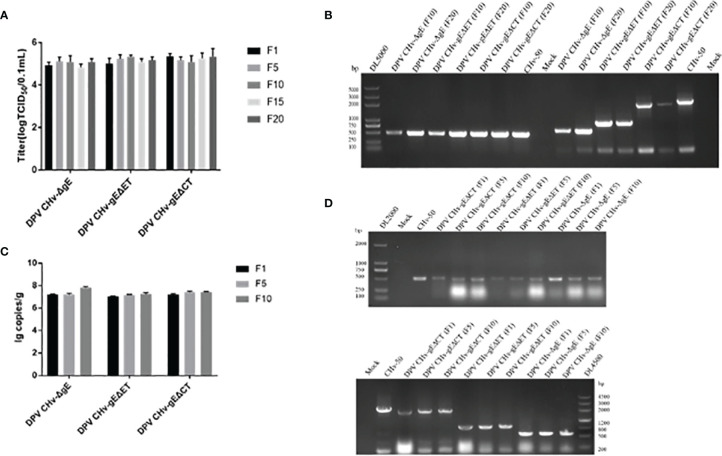
**(A)** Mutant viruses at passages 1st, 5th, 10th, 15th, and 20th were collected to infect DEFs at a 1 MOI. The cell samples were collected for virus copy number detection, *P*>0.05. **(B)** The DNA of the 10th and 20th generation mutant viruses was extracted from DEFs, respectively, and then the primers for *UL30* gene identification and *gE* gene identification primers were used for PCR identification. **(C)** Mutant viruses at passages 1st, 5th, and 10th were collected to infect ducklings at a 1 MOI. After 7 days of infection, the ducklings were slaughtered, 1 g of liver tissue was taken out, and the viral genome in the liver tissue was extracted for copy number determination, *P*>0.05. **(D)** DNAs of the 1st, 5th, and 10th generation mutant viruses were extracted from liver tissue, respectively, and PCR identification was performed with *UL30* gene identification primers and *gE* gene identification primers, respectively.

To evaluate the genetic stability of deletion mutant viruses *in vitro*, the viral titers of DPV CHv-gEΔET and DPV CHv-gEΔCT deletion mutant viruses at passages 1, 5, 10, 15, and 20 after infection in DEFs were detected. As shown in **Figure 4A**, the titers of deletion mutant viruses at different passages were all around 10^5^ TCID_50_/0.1 mL, with no significant difference (*P*>0.05). The virus titers of deletion mutant viruses were stable after serial passage on DEFs. DNA of passage 10 and 20 deletion mutant viruses were extracted and identified by PCR as described in Methods. As shown in **Figure 4B**, the *UL30* gene fragments from the DPV CHv-gEΔET and DPV CHv-gEΔCT mutant viral DNAs after serial passages were identified by the UL30 primers. The target gene fragments amplified from the DPV CHv-gEΔET and DPV CHv-gEΔCT mutant viral DNAs after gE deletion primers identified serial passages, and the sizes were in line with expectations. The above results show that after the serial passage of DPV CHv-gEΔET and DPV CHv-gEΔCT mutant viruses in DEFs, the deleted genes in the viral genome will not undergo reverse mutation and stably inherited *in vitro*.

**Figure 5:**
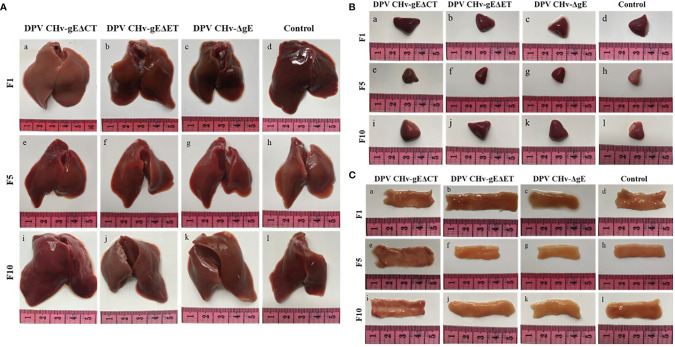
Pathological lesions of ducklings after challenge with the mutant virus at passages 1, 5, and 10. **(A)** Liver; **(B)** Spleen; **(C)** Duodenum.

To assess the genetic stability of deletion mutant viruses *in vivo*, the mortality of DPV CHv-gEΔET and DPV CHv-gEΔCT deletion mutant virus in ducklings after serial passage was detected, and the disease and virus infection in different organs were detected. As shown in **Table 2**, after the DPV CHv-gEΔCT group was infected with ducklings, only a small number of deaths occurred in the 2nd, 5th, and 6th generations, while the DPV CHv-gEΔET group and DPV CHv-ΔgE group were the same as the Mock group, no deaths occurred. The liver, spleen, and duodenum of ducklings infected with different generations of mutant deletion virus were necropsied to observe the lesions. After the ducklings were infected with DPV CHv-gEΔCT, only the duodenal mucosa showed slight hemorrhage, and other organs showed slight hemorrhage. No lesions were observed in all organs in the DPV CHv-gEΔET group and the DPV CHv-ΔgE group, as in the Mock group (**Figure 5**). The viral DNA in the liver of ducks infected with 1, 5, and 10 generations of deletion mutant virus was extracted, and PCR identification and quantitative detection of the virus content were carried out. As with the *in vitro* detection, all the target bands were in line with expectations, and the virus in the liver of different generations mutant virus loads was stable between 10^7^ ~ 10^8^ copies/g (**Figures 4C, D**). The above results show that after the DPV CHv-gEΔET and DPV CHv-gEΔCT mutant viruses are serially passaged in ducklings, the deleted genes in the viral genome will not undergo reverse mutation and can be stably inherited *in vivo*.

**Table 2:** The number of death ducklings for each group.

Group	F1	F2	F3	F4	F5	F6	F7	F8	F9	F10
DPV CHv-gEΔET	0/5	0/5	0/5	0/5	0/5	0/5	0/5	0/5	0/5	0/5
DPV CHv-gEΔCT	0/5	1/5	0/5	0/5	1/5	2/5	0/5	0/5	0/5	0/5
DPV CHv-ΔgE	0/5	0/5	0/5	0/5	0/5	0/5	0/5	0/5	0/5	0/5
Control	0/5	0/5	0/5	0/5	0/5	0/5	0/5	0/5	0/5	0/5

The numerator is the mortality numbers, and the denominator is the number of ducks challenged with viruses.

### Safety of DPV CHv-gEΔET and DPV CHv-gEΔCT

**Figure 6:**
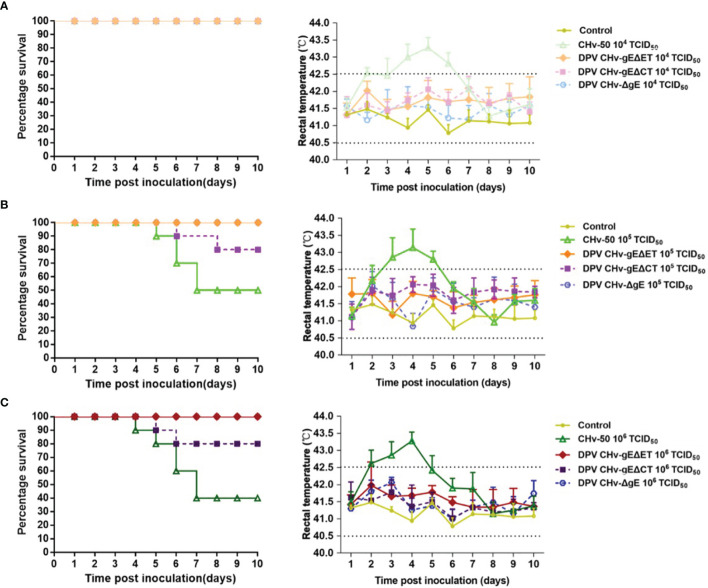
Survival percentage (left) and rectal temperatures (right) of ducklings. **(A)** Inoculated with a dose of 10^4^ TCID_50_ of mutant viruses; **(B)** Inoculated with a dose of 10^5^ TCID_50_ of mutant viruses; **(C)** Inoculated with a dose of 10^6^ TCID_50_ of mutant viruses.

The body temperature of most ducklings inoculated with parental virus CHv-50 will rise to above 43°C. The body temperature of ducklings in the 10^4^ TCID_50_ groups reached the peak on the 5th day, and the body temperature of the ducklings in the 10^5^ TCID_50_ and 10^6^ TCID_50_ groups both reached the peak on the 4th day. After reaching the peak, it will drop to the normal body temperature range of 40.5 ~ 42.5°C (**Figure 6**). 6 ~ 7 days is the peak period of death, and the mortality of different inoculation dose groups is distributed in a gradient. The ducklings in the 10^4^ TCID_50_ group did not die, and the ducklings in the 10^5^ TCID_50_ group had a mortality rate of 50%, and 1, 2, 2, died on days 5, 6, and 7, respectively. Ducklings in the10^6^ TCID_50_ group had a mortality rate of 60%, 1, 1, 2, and 2 died on days 4, 5, 6, and 7, respectively (**Figure 6**). The DPV CHv-gEΔET group, DPV CHv-gEΔCT group, DPV CHv-ΔgE group were the same as the MEM group, the body temperature of the different dose groups after inoculation of the ducklings always fluctuated within the normal range within 10 days. Only DPV CHv -gEΔCT group died at 10^5^ TCID_50_ and 10^6^ TCID_50_ doses, and the mortality rate was 20% (**Table 3**). No deaths occurred within 10 days in the DPV CHv-gEΔET group, DPV CHv-gEΔCT group, DPV CHv-ΔgE group, and MEM group. The above results indicated that both ET and CT lacking gE would reduce the pathogenicity of the virus to ducklings, while the pathogenicity of the ET virus lacking gE was more significantly reduced to ducklings.

**Table 3:** Mortality statistics of ducks.

Group	Challenge dose (TCID_50_)		
	10^4^	10^5^	10^6^
CHv-50	0/10	5/10	6/10
DPV CHv-gEΔET	0/10	0/10	0/10
DPV CHv-gEΔCT	0/10	2/10	2/10
DPV CHv-ΔgE	0/10	0/10	0/10
MEM	0/10	0/10	0/10

The numerator is the mortality numbers, and the denominator is the number of ducks challenged with viruses.

### Immunogenicity of DPV CHv-gEΔET

**Figure 7:**
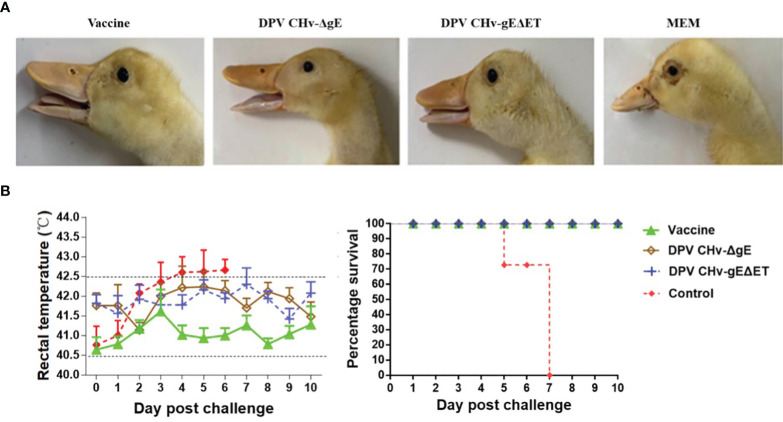
The ducklings were challenged after immunization, and the clinical symptoms of the ducklings after the challenge were observed. **(A)** Eyelid condition. **(B)** Rectal temperature (left) and survival rate (right).

The clinical symptoms of the ducklings in different immunization groups after the challenge were observed. Only the ducklings in the control group began to be lethargic, poor in appetite, tearing, and the feathers around the eyelids formed eye circles on the 4th day after the challenge. As shown in **Figure 7**, the body temperature of the ducklings in different immunization groups was measured after the challenge, the body temperature of the control group increased, and the body temperature exceeded 42.5°C on the 4th day after the challenge, and then the body temperature continued to be high until all died. The body temperature of the DPV CHv-ΔgE group and DPV CHv-gEΔET group was slightly higher than that of the vaccine group, but both fluctuated within the normal range. Statistics on the death of ducklings in different immunization groups after challenge, the control group began to die on the 5th day, and the 7th day was the peak period of death, and all died within 7 days, while the DPV CHv-ΔgE group, DPV CHv-gEΔET group like the vaccine group, all the ducklings were survived, indicating that the ducklings immunized with DPV CHv-ΔgE and DPV CHv-gEΔET could resist the challenge of virulent DPV CHv by 100%.

**Figure 8:**
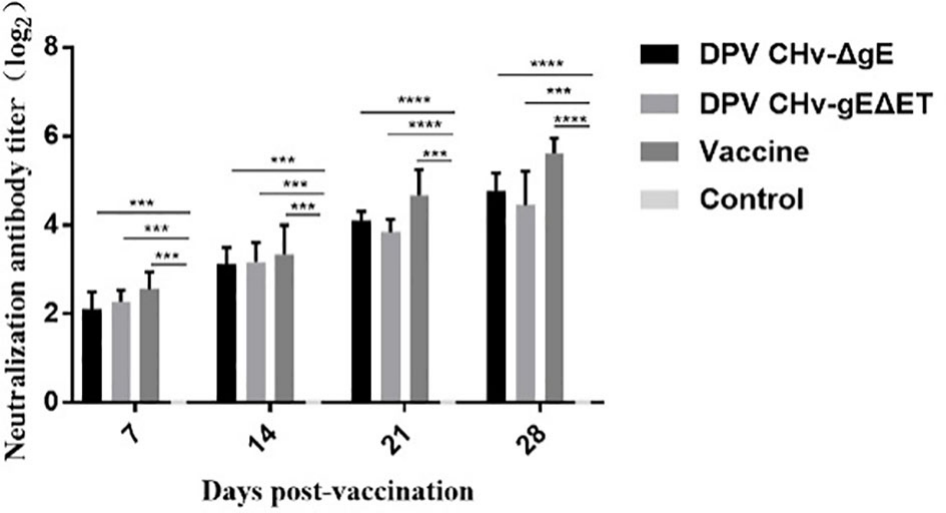
The determination of neutralizing antibody titer in ducks of different groups after immunization. ***P< 0.001, ****P< 0.0001.

Neutralizing antibodies were detected in the sera of ducklings in different immunization groups. Neutralizing antibodies could be detected in the DPV CHv-ΔgE group, DPV CHv-gEΔET group, and vaccine group on the 7th day after immunization, and then the antibody levels gradually increased. On the 28th day after immunization, the neutralizing titers in the sera of ducklings in different immunization groups DPV CHv-ΔgE group, DPV CHv-gEΔET group, and vaccine group reached 2^4.8^, 2^4.4,^ and 2^5.6^ (**Figure 8**), respectively, there was no significant difference in valence (*P*>0.05), but both were extremely significantly higher than those in the control group (*P*<0.001, *P*<0.0001), indicating that DPV CHv-ΔgE and DPV CHv-gEΔET could stimulate ducklings to produce significant neutralizing antibodies.

## Discussion

In *alphaherpesviruses*, *gE* is an important virulence gene that affects the virus’s virulence. Selecting the deletion of the *gE* gene can reduce the virulence of the virus without affecting the immunogenicity and growth ability of the virus (34, 35). Virulence genes such as gE and gI have various degrees of insertion, deletion, and base substitution mutations, resulting in the emergence of highly toxic and lethal PRV mutant strains (36–39). The PRV mutant strain ZJ01, with 2 amino acid insertions in its gE sequence, caused all 14/80-day-old pigs to die (40). Two recombinant viruses, rZJ01-LA/gEI and rLA-ZJ01/gEI, were generated by exchanging the *gE* and *gI* genes of the LA strain and the ZJ01 strain. It was found that rLA-ZJ01/gEI exhibited higher virulence than its parental virus rLA, indicating that changes in the *gE* gene were part of the reason for the increased virulence of PRV strains in recent years (41). The bacterial artificial chromosome (BAC) of DPV is an effective tool for the study of duck plague virus, an infectious clone of a virulent strain of DPV CHv (DPV CHv-BAC-GS1783) was previously constructed in our laboratory and used in this study to generate mutant virus DPV CHv-ΔgE, DPV CHv-gEΔET and DPV CHv-gEΔCT (29, 30, 42, 43). It should be further mentioned that the above mutant virus lacks gE and different functional regions of gE and lacks miniF on its basis. Otherwise, the deletion-mutant virus cannot infect ducks or be used as a live vaccine. The *gE* gene of DPV has been confirmed to play an important role in the assembly of virions, with only minor damage to virus replication. In this study, the dynamic growth curve of the mutant virus after gE deletion in different regions was determined, which also confirmed this conclusion. By measuring the *in vitro* proliferation of DPV CHv-gEΔET and DPV CHv-gEΔCT mutant viruses on DEFs, both mutant viruses were found to reduce virus titers on host cells, but to a lesser extent. However, further research is needed on the specific functions of gE ET and gE CT.

Vaccine immunization is an important means of preventing and controlling viral diseases. Recombinant vector vaccines have become one of the hotspots in vaccine research due to their unique advantages. There are many non-essential regions in the DPV genome (1, 3), such as the *gE/gI* genes (44, 45), which can be used as insertion sites for foreign genes. Therefore, DPV attenuated/attenuated strains have the advantage of being used as multiplex recombinant live vaccine carriers in addition to being used as DPV vaccines. Many researchers have expressed the immunogenic genes of Avian Influenza virus, duck Tembusu virus, duck hepatitis virus, chicken infectious bronchitis virus, and Newcastle disease virus using traditional attenuated vaccine strains (46–52). DPV immunized ducks with recombinant Avian Influenza virus can produce better protective effects. Immunized chickens can also quickly produce immune protection. The virus cannot replicate efficiently in chickens, so the safety is high. Therefore, the analysis of DPV-related genes, especially the functions of virulence-related genes similar to gE, is of great significance for the prevention and control of DPV and immune purification and promoting the application of DPV in avian multivalent live vector vaccine vectors.

The genetic stability of mutant virus is one of the important contents of mutant virus research. The stability of the mutant virus is evaluated mainly through the continuous passage of the mutant virus *in vitro* and *in vivo*. We serially passaged the mutant viruses DPV CHv-gEΔET and DPV CHv-gEΔCT on DEFs and in ducklings, respectively, and measured the virus titer, whether the deleted gene was restored, and the pathogenic changes in susceptible animals. Found that the missing gene sequence of the mutant virus has not recovered, nor has the virulence returned, indicating that the mutant virus has good genetic stability, which is the premise for subsequent studies of mutant viruses as multivalent live vector vaccines or attenuated vaccine vectors. We grouped the mutant viruses DPV CHv-gEΔET and DPV CHv-gEΔCT according to the challenge dose gradient setting and conducted a pathogenicity study in 14-day-old ducklings, and preliminary analysis was made from body temperature, death, and antibody production after challenge. The results showed that the body temperature of the ducklings in the parental virus CHv-50 10^5^ TCID_50_, 10^6^ TCID_50_ groups increased and exceeded the normal body temperature range 3 to 4 days after the challenge, and the mortality rate showed a gradient difference according to the challenge dose. The body temperature of DPV CHv-gEΔCT fluctuated within the normal range, and the mortality rates were 20% (10^6^ TCID_50_), 20% (10^5^ TCID_50_), and 0% (10^4^ TCID_50_), respectively. The body temperature of DPV CHv-gEΔET and DPV CHv-ΔgE in each dose group, fluctuated within the normal range, and no death occurred. DPV-specific serum antibodies could be detected in the ducklings on the 10th day after inoculation (data not shown). The above data show that gE deletion of ET or CT will reduce the pathogenicity of the virus. The pathogenicity of the mutant virus to ducklings after gE deletion of ET is significantly reduced, and the ducklings can be induced to produce specific serum antibodies. It further shows that DPV *gE* is also an important virulence gene, and the gE ET is an important region associated with virulence.

On the basis that the DPV CHv-gEΔET mutant virus has been proved to have stable inheritance, significantly reduce virus virulence, and induce DPV-specific serum antibodies in ducklings, further research on subsequent vaccines was carried out. In this study, the DPV CHv-gEΔET mutant virus was used to study the immune efficacy of the DPV CHv-gEΔET mutant virus by selecting the live duck plague vaccine that is widely used in China as a comparison. In the challenge protection trial, a lethal dose of CHv was administered after immunization, and no death and clinical symptoms were found in the DPV CHv-gEΔET, DPV CHv-ΔgE, and vaccine groups, and the challenge protection rate was 100%, the control group all died. In addition, judging whether the potential to develop into a vaccine is related to the challenge protection rate and observing the changes in antibody levels after immunization. Whether the body can produce high antibodies for a long time after vaccination is very important to fight viral infection. It is also one of the criteria for evaluating the quality of vaccines. Through neutralizing antibody detection, it was found that ducklings could produce certain neutralizing antibodies on the 7th day after immunizing with DPV CHv-ΔgE or DPV CHv-gEΔET mutant virus, and the antibody level on the 7th to 28th day after immunization rising. The levels of neutralizing antibodies produced after immunizing ducklings with the two mutant viruses were similar, significantly higher than those in the Mock group but slightly lower than those in the vaccine group. The above shows that the deletion of the *gE* gene or the deletion of gE ET in DPV reduces the virus’s virulence and stimulates the ducklings to produce a similar level of the humoral immune response, which can provide the same protection to the immunized animals. In this study, we lacked the detection of cellular immunity, and perhaps the reason for the immune protection of the DPV CHv-gEΔET mutant virus could be further explained by the study of cellular immunity.

It is well known that gE and gI function as dimers in *alphaherpesviruses* (10, 53). Previous studies in our laboratory have also demonstrated that gE and gI in DPV can form dimers, and amino acids 1-279 of gI can locate gE from the endoplasmic reticulum to the Golgi O-glycosylation modification (data unpublished). However, whether the complex position between gE and gI in DPV is also in the gE ET region, and the specific amino acid sites in gE that affect the complex formation with gI are currently being screened. The current research results and subsequent related research will provide a corresponding theoretical basis for the prevention and control of DPV and duck plague purification and promote the application of DPV in avian multivalent live vector vaccine vectors.

## Data Availability Statement

The datasets presented in this study can be found in online repositories. The names of the repository/repositories and accession number(s) can be found in the article/supplementary material.

## Ethics Statement

The experimental animal protocol was approved by the Ethics and Animal Welfare Committee of Sichuan Agricultural University and carried out following the Chinese version of the Guide for the Care and Use of Laboratory Animals.

## Author Contributions

Conceptualization, YN and AC. Methodology, YN and YH. Software, YN and QY. Validation, MW. Formal analysis, YN. Investigation, YN. Resources, AC, MW, SC, DZ, ML, QY, YW, XZ, SZ, JH, BT, RJ, XO, SM, QG, DS, YY, and LZ. Data curation, YN. Writing-original draft preparation, YN. Writing-review and editing, AC. Visualization, BT, RJ, ML, QY, and DS. Supervision, YN and AC. Project administration, MW and AC. Funding acquisition, MW, AC, RJ, SC, DZ, and ML. All authors have read and agreed to the published version of the manuscript.

## Funding

This work was supported by the National Natural Science Foundation of China (32072894), the China Agriculture Research System of MOF and MARA, and the Sichuan Veterinary Medicine and Drug Innovation Group of China Agricultural Research System (SCCXTD-2020-18).

## Conflict of Interest

The authors declare that the research was conducted in the absence of any commercial or financial relationships that could be construed as a potential conflict of interest.

## Publisher’s Note

All claims expressed in this article are solely those of the authors and do not necessarily represent those of their affiliated organizations, or those of the publisher, the editors and the reviewers. Any product that may be evaluated in this article, or claim that may be made by its manufacturer, is not guaranteed or endorsed by the publisher.
